# STEMIs and a Closer Look at MINOCA During the COVID-19 Pandemic

**DOI:** 10.1016/j.jscai.2022.100372

**Published:** 2022-05-19

**Authors:** Mirvat Alasnag, Binita Shah, Giulia Botti, Sarah Zaman, Alaide Chieffo

**Affiliations:** a Cardiac Center, King Fahd Armed Forces Center, Jeddah, Saudi Arabia; b Division of Cardiology, Department of Medicine, NYU Grossman School of Medicine, New York, New York; c Interventional Cardiology Unit, IRCCS San Raffaele Scientific Institute, Milan, Italy; d Department of Cardiology, Westmead Hospital, Sydney, Australia

**Keywords:** COVID-19, outcomes, sex differences, STEMI

The universal definition of myocardial infarction (MI) differentiates between type 1 and type 2 MI.^1^ Type 1 generally refers to acute atherothrombotic disease that is precipitated by atherosclerotic plaque disruption (rupture or erosion). Type 2 occurs as a result of an imbalance in oxygen supply in the setting of greater-than-usual demand. At the onset of COVID-19 pandemic, much of the focus was on type 1 MI, which was observed to be associated with an unusually large thrombus burden, and its management, including door-to-balloon times, hemodynamic support in concomitant shock, and cardiopulmonary resuscitation during cardiac arrest. Early reports noted delays in presentation, concern over pathways advocating the use of thrombolytic therapy over primary percutaneous intervention (PPCI), and controversy over how best to implement advanced cardiac life support.^2^, ^3^, ^4^, ^5^ Over the subsequent 2 ​years, the high risk of thrombosis in COVID-19, profound hypoxemia, and the severe systemic inflammatory response confounded our understanding and management of ST-segment elevation MI (STEMI) and cardiogenic shock. Questions that arose included whether those with a hypercoagulable state benefit from PPCI vs potent invasive pharmacotherapy and whether MI with nonobstructive coronary artery (MINOCA) is, in fact, type 2 MI due to COVID-19 complications.

The North American COVID-19 STEMI (NACMI) registry was an opportunity to capture the patterns of presentation during COVID-19 as well as temporal changes in practice and outcomes.^6^ In North America, PCI remains the predominant revascularization strategy in the majority of patients, with two-third of them achieving the recommended door-to-balloon time of less than 90 ​minutes. Over time, survival approximated the pre-COVID era, with higher mortality rates in unvaccinated patients. Initially, there was a notable higher incidence in minority groups with less favorable outcomes; worse economic conditions and a higher prevalence of comorbidities were among the postulated explanations. During the most recent wave of the pandemic, the racial and ethnic breakdown of patients in the NACMI registry once again reflected prepandemic data, with a predominance in Caucasians.^7^^,^^8^

The current substudy by Quesada et al^9^ evaluated sex differences observed in those with STEMI and COVID-19. The registry data noted what we have long observed, before the pandemic, that women were older and more likely to have risk factors like diabetes or comorbidities like stroke; however, compared with prepandemic times where chest pain was the most common presenting symptom in both women and men, we now observe dyspnea to be the most common presenting symptom in women. What is most striking, however, is the lack of an identifiable culprit vessel in 21% of STEMIs, markedly higher than the proportion of STEMIs without concomitant COVID-19 in which it was observed. Indeed, the incidence of MINOCA in STEMI without COVID-19 is only 3%-6%.^10^ A limitation of the NACMI registry is that it can only highlight this interesting epidemiology but cannot inform us further on the potential causes for this high rate of MINOCA. Women have long been more than twice as likely as men to have MINOCA, and this did not differ in the current analysis (MINOCA was present in 33% of women vs 18% of men; *P* ​< ​.001). As such, women were more likely to receive medical therapy alone, whereas men were more likely to receive PPCI. Intracoronary imaging, provocative testing, cardiac magnetic resonance imaging, and thrombophilia assays would have been valuable in discerning the most common cause of MINOCA in COVID-19 and may need to be captured in future registry data (Figure 1). In-hospital mortality was also markedly high at 29% but did not differ by sex (33% for women and 27% for men; *P* ​= ​.22). This, however, was significantly higher than that in the pre-COVID-19 pandemic era. In-hospital mortality correlated with moribund conditions and clinical presentations such as cardiogenic shock, age >66 ​years, prior stroke, and pulmonary infiltrates. This finding suggests that comorbidities had a large impact on survival.

**Figure 1:**
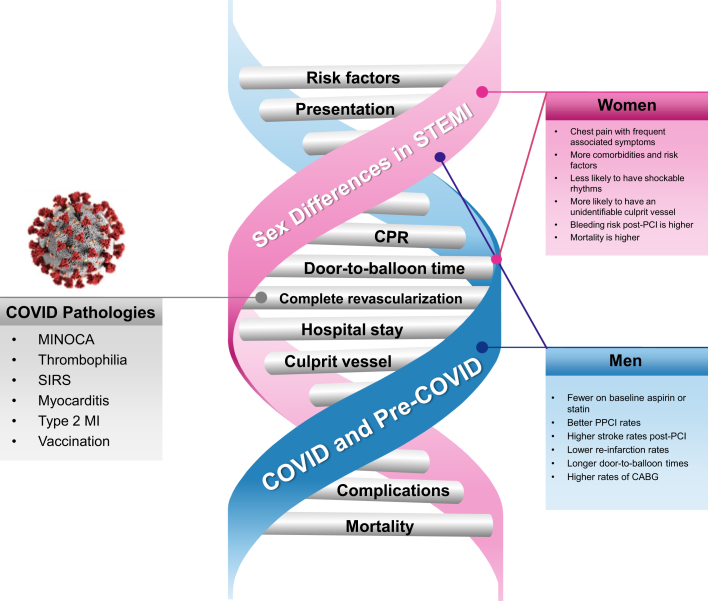
Unique features of STEMI and MINOCA in men and women with COVID-19 infection. CABG, ​coronary artery bypass grafting; CPR, ​cardiopulmonary resuscitation; MI, ​myocardial infarction; MINOCA, ​myocardial infarction with nonobstructive coronary arteries; PCI, ​percutaneous coronary intervention; PPCI, primary PCI; SIRS, ​systemic immune response syndrome; STEMI, ST-segment elevation MI.

Although NACMI lacks adjudication of outcomes, it is a representation of disease patterns and practice trends. The NACMI sex substudy provides an incentive to understand MINOCA further and press clinicians to investigate these cases more accurately. Furthermore, as the data in the registry continue to grow, an analysis of outcomes by race/ethnicity and sex may provide additional insight into any potential disparities of care. Finally, additional analyses of temporal trends with respect to the vaccinated and unvaccinated populations, the differing variants leading to COVID-19, and/or patterns in delivery of cardiac services during surge peaks or as restrictions are lifted may also be informative. Despite the limitations of this registry, it is important for the interventional cardiology community to continue to understand the changing landscape of STEMI in the setting of COVID-19 and adapt accordingly.

## Declaration of competing interest

None of the authors have any relevant financial disclosures.
